# Comprehensive analysis of DTYMK in pan-cancer and verification in lung adenocarcinoma

**DOI:** 10.1042/BSR20221170

**Published:** 2022-10-18

**Authors:** Yue Zhang, Hao Wang, Ying Liu, Jing Yang, Xiaoxiao Zuo, Meilian Dong, Zhigang Zhang, Yonggang Shi, Xubin Deng, Yaoyong Lv

**Affiliations:** 1 Department of Radiation Oncology, The First Affiliated Hospital of Zhengzhou University, Zhengzhou, Henan, China; 2 Department of Oncology, The Six Affiliated Hospital of Guangzhou Medical University, Qingyuan People’s Hospital, Qingyuan, Guangdong, China; 3 Affiliated Cancer Hospital and Institute of Guangzhou Medical University, Guangzhou, China; 4 Department of Oncology (Section 3), Gaozhou People’s Hospital, Gaozhou, Guangdong, China

**Keywords:** DTYMK, lung adenocarcinoma, pan-cancer

## Abstract

Previous documents have reported that the deoxythymidylate kinase (DTYMK) genes were involved in the progression of cancers. However, its significance in the analysis of pan-cancer and specific molecular mechanism were still poorly understood. In the present study, we conducted a comprehensive study of the DTYMK gene associated with its clinical relevance across a broad-spectrum of human tumors. In addition, association among DTYMK gene and tumor immunogenic features was also explored. Considering the results of pan-cancer analysis, the specific tumor lung adenocarcinoma (LUAD) was chosen to further study the DTYMK-induced signaling pathways and intercellular communications in tumor progression. Our findings demonstrated that DTYMK may be a new biomarker for the prognosis and immunotherapy in various cancers. Importantly, DTYMK was expected to be a guiding marker gene for clinical prognosis and tumor personalized therapy in LUAD.

## Introduction

Lung cancer is one of the most prevalent malignancies across the globe [1]. It is categorized into small cell lung carcinoma (SCLC), which is attributable to 15% of cases, and non-small cell lung carcinoma (NSCLC), which is attributed to the remaining 85% [2]. There are three subtypes of NSCLC: large cell carcinoma, lung squamous cell carcinoma, and adenocarcinoma (LUAD) [3]. The most prevalent histological type of lung cancer is LUAD representing 40% of all cases of the disease [4]. The overall survival probability of people who have LUAD is still unsatisfactory, despite the advancements that have been made in diagnosis and therapy over the last several years [5]. According to the available statistics, the mean survival probability over 5 years is below 20%. The enhancement of treatment outcomes is hampered by a lack of adequate knowledge of the basic process underlying LUAD [6]. Therefore, it is of the utmost importance to make more progress in explaining tumor onset and discovering novel biological markers to improve prognosis.

The synthesis of DNA is a necessary step in the process of cell replication, particularly in tumor cells. In the therapeutic intervention of many forms of cancer, pharmacological drugs that target the production and metabolism of deoxyribonucleoside triphosphate are often employed [7]. In the *de novo* pathway, thymidylate synthase is responsible for the methylation of deoxyuridine-5′-monophosphate, resulting in the production of deoxythymidine-5′-monophosphate (dTMP) [8]. dTMP is synthesized via the salvage pathway when thymidine is phosphorylated by thymidine kinase. Deoxythymidylate kinase (DTYMK) is an enzyme that has the ability to catalyze the dTMP phosphorylation, which ultimately results in the formation of dTDP [9]. In addition, it is the first merging phase of both the *de novo* and salvage pathways in the generation of dTTP, an essential component for DNA synthesis. According to previous research, knockdown of DTYMK blocked this pathway, resulting in a reduction in the product dTDP as well as a buildup of the substrate dTMP [10]. Nevertheless, the biological role of DTYMK in pan-cancer, especially in LUAD is still poorly unknown.

In this research, the relative expression and prognostic significance of the DTYMK gene in pan-cancer were investigated. An investigation was conducted to determine whether there is a correlation between the levels of DTYMK gene expression and TME, stemness, and immunological subtypes. Additionally, the involvement of the carcinogenic gene DTYMK in LUAD was highlighted in this research.

## Materials and methods

### Analysis of DTYMK in pan-cancer

We measured the DTYMK expression differences between tumor and normal tissue samples premised on data from tumor cell lines retrieved from the Cancer Cell Line Encyclopedia (CCLE) database, as well as data from normal tissues retrieved from the Genotype-Tissue Expression (GTEx) database and The Cancer Genome Atlas (TCGA). An online software HOME for Researchers (https://www.home-for-researchers.com/static/index.html#/) was used to perform the analysis.

With the use of the human protein atlas (HPA) database (https://www.proteinatlas.org/), the DTYMK protein expression levels, as well as its distribution in LUAD tissues, were displayed.

### Survival analysis

By utilizing the clinical data contained in the TCGA database, we evaluated the patients’ survival and prognoses interms of overall survival (OS) and progression-free interval (PFS).The link between DTYMK expression and patients’ prognoses was displayed utilizing forest plots. Lastly, survival analysis was performed utilizing Kaplan–Meier curves, which were applied to the data from specific malignancies that had statistical significance.

### Immune infiltration analysis, immune checkpoint, TMB, and MSI status analysis

The RNA-sequencing expression profiles and relevant clinical data for DTYMK were downloaded from the TCGA dataset (https://portal.gdc.com). Analysis was conducted to examine the link between the expression of DTYMK and the immune infiltration of distinct malignancies. PDCD1LG2, LAG3, CTLA4, PDCD1, HAVCR2, CD274, IDO1, and SIGLEC15 are the eight genes whose expression levels were analyzed in order to derive a value for the immune checkpoint-related genes.

Tumor mutation burden (TMB) was based on the research presented in the publication titled “The Immune Landscape of Cancer,” which was written by Vesteinn Thorsson and his colleagues in 2018 [11]. Microsatellite instability (MSI) wasbased on an article titled “Landscape of Microsatellite Instability across 39 Cancer Types,” which was authored by Russell Bonneville and colleagues in 2017 [12].

The above analysis was performed with the online software HOME for Researchers (https://www.home-for-researchers.com/static/index.html#/).

### Single-cell RNA sequencing (scRNA-seq) analysis of DTYMK in LUAD

The scRNA-seq analysis was conducted with the online software Cancer SEA (http://biocc.hrbmu.edu.cn/CancerSEA/).

### Cell maintenance

Cell lines derived from human LUAD were procured from the China Center for Type Culture Collection (CCTCC). The cells were grown in PRMI/1640 media with 10% fetal bovine serum (FBS) and 1% penicillin-streptomycinin a humid chamber comprising 5% carbon dioxide. The temperature of the incubator was set at 37°C.

### MTT, colony formation, and cell cycle assays

Cells were placed into 96-well plates for the MTT assay. Following an incubation period of 24 h, 5 mg/l of MTT was introduced into each well. After this, the cells were allowed to continue to grow in the incubator for a further 6 h. The absorbance was then observed at 490 nm utilizing a Spectrophotometer after DMSO was added to each well.

Cells were put on to 6-well plates and incubated to perform the clone formation experiment. After 2 weeks had elapsed, the cells were fixed with paraformaldehyde before being stained with a solution of hematoxylin, and then we counted them using a microscope.

To conduct the cell cycle assay, we removed the cells from the growth plates and subsequently rinsed them three times using cold PBS. After that, the cells were fixed using ice-cold ethanol at a concentration of 70% throughout the night at 4°C. Afterward, an incubation with propidiumiodide containing RNase A was performed on the cells. The DNA content of labeled cells was analyzed with the use of FACS caliber flow cytometry (BD Biosciences).

### Quantitative reverse transcription-polymerase chain reaction (qRT-PCR) analysis

After extracting total RNA from the cultured cells with the aid of Trizol reagent (Invitrogen), they were subsequently reverse-transcribed to acquire stable cDNA utilizing PrimeScript™ RT reagent Kit containing gDNA Eraser (TaKaRa Bio). Next, we conducted qRT-PCR utilizing SYBR Premix Ex Taq II (TaKaRa Bio) in Quant Studio™ 3 real-time fluorescent quantitative PCR instrument (Thermo Fisher Scientific). To standardize the level of gene expression, the glyceraldehyde 3-phosphate dehydrogenase (GAPDH) was utilized as an internal control. The results were computed utilizing the 2^−ΔΔCt^ method, and the outcomes were reported as multiple changes in relation to GAPDH. Primers for DTYMK were as follows: forward primer 5′-CCGGTTCCCGGAAAGATCAAC-3′ and reverse primer 5′-TCCCAGCGATTTGCAGAAAAA-3′. Primers for GAPDH were: forward primer 5′-GGAGCGAGATCCCTCCAAAAT-3′ and reverse primer 5′-GGCTGTTGTCATACTTCTCATGG-3′.

### Western blot assay

To perform Western blotting, the protein specimens were first loaded on to a PVDF membrane, and then they were placed in primary antibodies at a 1:500 dilutionand incubated at 4°C throughout the night. After that, the membranes were incubated for one hour at ambient temperature with HRP-conjugated rabbit or mouse and subsequently developed using a chemiluminescence reagent.

### Statistical analysis

All quantitative data are represented as mean ± standard deviation (SD). The Student’s *t*-test and the one-way analysis of variance (ANOVA) were applied to evaluate the data and detect statistically significant differences. *P*-value < 0.05 was established as a criterion of statistical significance.

## Results

### Abnormal expression and prognostic analysis of DTYMK in pan-cancer

**Figure 1:**
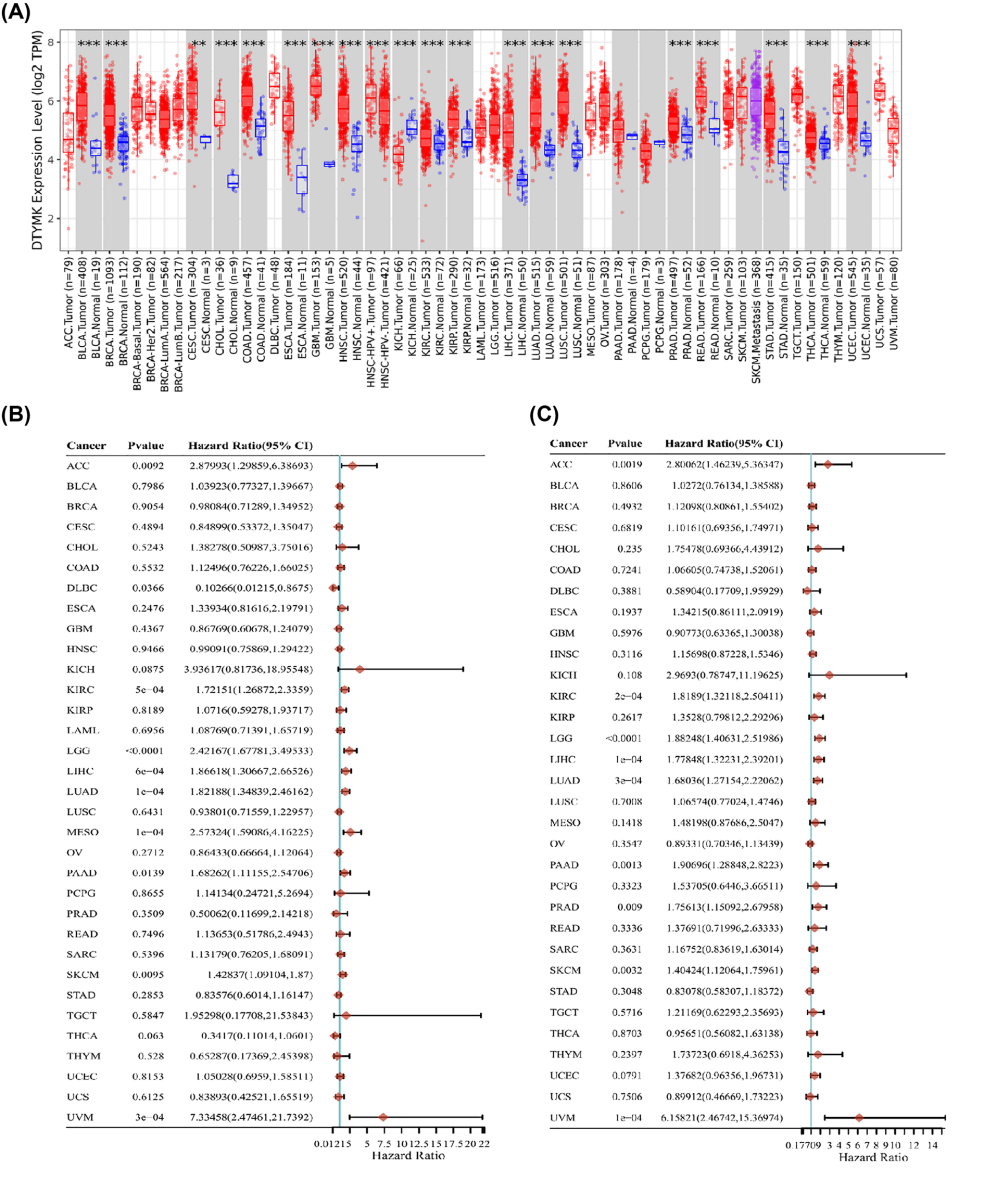
Aberrant expression and prognostic analysis of DTYMK in pan-cancer (**A**) DTYMK expression in different cancers from TCGA based on TIMER2.0 (**P<0.01, ***P<0.001). (**B,C**) Forest plots for DTYMK expression levels and overall survival and progression-free survival in pan-cancer. The hazard ratios (HRs) and 95% confidence intervals are shown. HR < 1 and HR > 1 represent a low and high risk, respectively.

First, we employed the TIMER2.0. software to analyze DTYMK expression level in pan-cancer by mining the TCGA database. The findings illustrated that DTYMK expression level was elevated in UCEC, CESC, THCA COAD, READ, KIRP, KIRC, LIHC, HNSC, LUAD, GBM, LUSC, ESCA, PRAD, CHOL, STAD, BRCA, and BLCA. However, the level of DTYMK expression was down-regulated in KICH (Figure 1A). In addition to this, we investigated whether there was a link between DTYMK expression and the OS rate for each of the 33 distinct cancers in TCGA. The forest plots illustrated that up-regulated expression of DTYMK was linked to the unfavorable OS in UVM, DLBC, PAAD, LGG, SKCM, MESO, LUAD, LIHC, KIRC, and ACC (Figure 1B). Additionally, we discovered that up-regulated expression of DTYMK was related to shorter PFS in UVM, LUAD, PRAD, LIHC, SKCM, PAAD, LGG, KIRC, and ACC (Figure 1C). Based on these findings, we also evaluated the link between the expression of DTYMK and clinical stages in these types of cancer by analyzing the TISIDB database (http://cis.hku.hk/TISIDB/). The data illustrated that DTYMK expression was considerably linked to clinical stages in LUSC, LIHC, KIRP, KIRC, LUAD, KICH, and ACC (Supplementary Figure S1A).

Collectively, the above findings show that DTYMK expression was up-modulated in numerous malignancies, suggesting that DTYMK may have an oncogenic function in these cancers.

### Relationship between DTYMK expression levels and immune infiltration, immune checkpoint, TMB, and MSI status in pan-cancer

**Figure 2:**
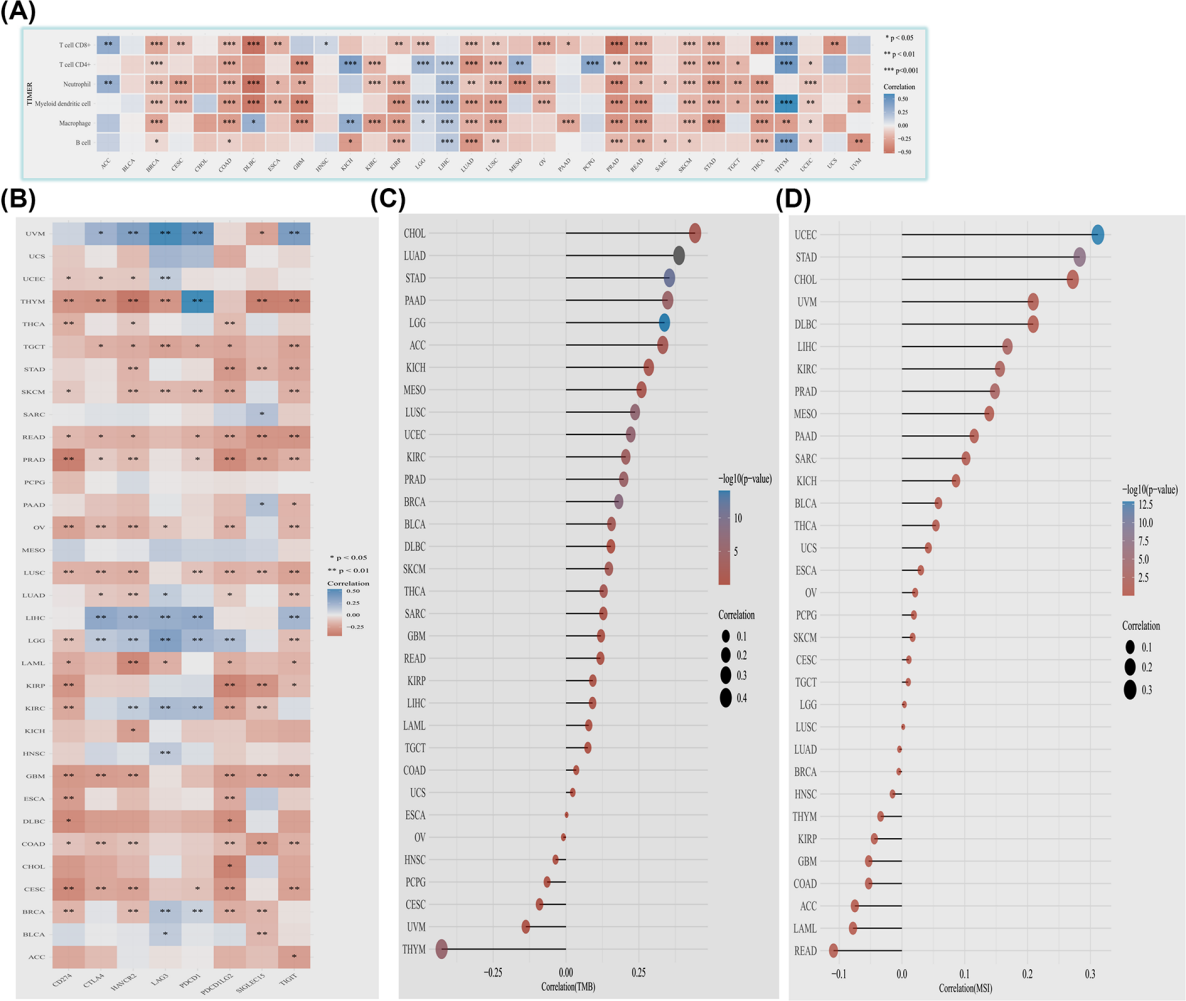
Association between DTYMK expression level and immune infiltration level, immune checkpoint, TMB, and MSI status in pan-cancer (**A**) Correlation between DTYMK expression level and immune infiltrating cells in pan-cancer. (**B**) Correlation between DTYMK expression level and key immune checkpoints in pan-cancer was indicated. (**C**) Correlation between DTYMK expression level and TMB in pan-cancer. (**D**) Correlation between DTYMK expression level and MSI status in pan-cancer. *P<0.05, **P<0.01, ***P<0.001.

First, we examined the possible link between DTYMK expression and the immune infiltration degree in pan-cancer. The TIMER approach was utilized to investigate the relationship that exists between DTYMK expression and immune-infiltrating cells. The data illustrated that DTYMK was notably correlated with in filtrating dendritic cells, B cells, neutrophils, CD4+ T cells, macrophages, and CD8+ T cells in most kinds of cancer, except in BLCA, CHOL, HNSC, KICH, LGG, PAAD, PCPG, THYM, UCS, and UVM (Figure 2A and Supplementary Table S1).

Afterward, we evaluated the link between DTYMK expression and key immune checkpoints (CD274, CTLA-4, HAVCR2, LAG3, PDCD1, PDCD1LG2, SIGLEC15, and TIGIT) expression levels in pan-cancer. As revealed by the findings, the DTYMK expression level was substantially correlated with one or more of these markers, except in MESO, PCPG, and UCS (Figure 2B and Supplementary Table S2).

We also analyzed the association between DTYMK expression level and TMB. We discovered that DTYMK expression level had a positive link to TMB in LUAD, LGG, STAD, BRCA, LUSC, UCEC, PAAD, PRAD, KIRC, SKCM, BLCA, ACC, THCA, CHOL, MESO, and KICH, but was negatively linked to TMB in THYM (Figure 2C and Supplementary Table S3).

Finally, we analyzed the association between DTYMK expression level and MSI status. We found a positive link between DTYMK expression level and MSI status in UCEC, STAD, PRAD, LIHC, and KIRC (Figure 2D and Supplementary Table S4).

### Association among DTYMK expression level and clinicopathological characters in LUAD

**Figure 3:**
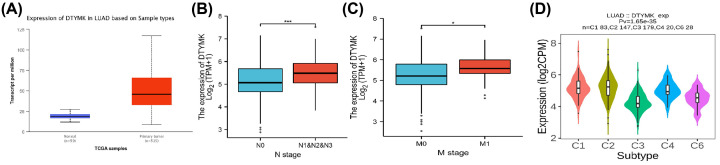
Association between DTYMK expression level and clinico-pathological characters in LUAD (**A**) The expression levels of DTYMK in LUAD tissues and normal tissues were demonstrated, respectively. Blue: normal tissues; Red: LUAD tissues. (**B**) Association between DTYMK expression level and node metastasis was indicated. Blue: no node metastasis; Red: node metastasis (N1&N2&N3). (**C**) Association of DTYMK expression level and distant metastasis was present. Blue: no distant metastasis; Red: distant metastasis (M1). (**D**) Association of DTYMK expression level and immune distinct types in LUAD. C1-C6 were immune distinct types were demonstrated, respectively. *P<0.05, **P<0.01, ***P<0.001.

We examined DTYMK expression and patient prognosis in LUAD. We assessed DTYMK expression in LUAD and normal lung samples by mining the TCGA database. The expression levelsof DTYMK were found to be considerably elevated in LUAD samples in comparison with normal samples, as illustrated in Figure 3A. Next, the association between clinical-pathological characters and DTYMK expression levels was analyzed. We revealed that DTYMK expression levels were significantly associated with node metastasis and distant metastasis, though there was no significance between DTYMK expression level and T stage. LUAD patients exhibiting elevated DTYMK expression levels tend to have high node metastasis and distant metastasis rate (Figure 3B,C).

There was a total of six immune distinct types that were discovered in solid tumors, which included C1 (wound healing), C2 (IFN-c dominant), C3 (inflammatory), C4 (lymphocyte depleted), C5 (immunologically quiet), and C6 (TGF-β dominant). The findings illustrated favorable prognoses among patients with C3 and C5 immune types, whereas those with C4 and C6 exhibited significant survival risks. With the use of the TISIDB database, we analyzed the association between DTYMK expression level and the immune subtype. Elevated DTYMK expression level was discovered in LUAD patients belonging to subtypes C1, C2, C4, and C6, illustrating that DTYMK might be implicated in the promotion of tumor progression (Figure 3D).

### Association between DTYMK expression level and prognostic in LUAD

**Figure 4:**
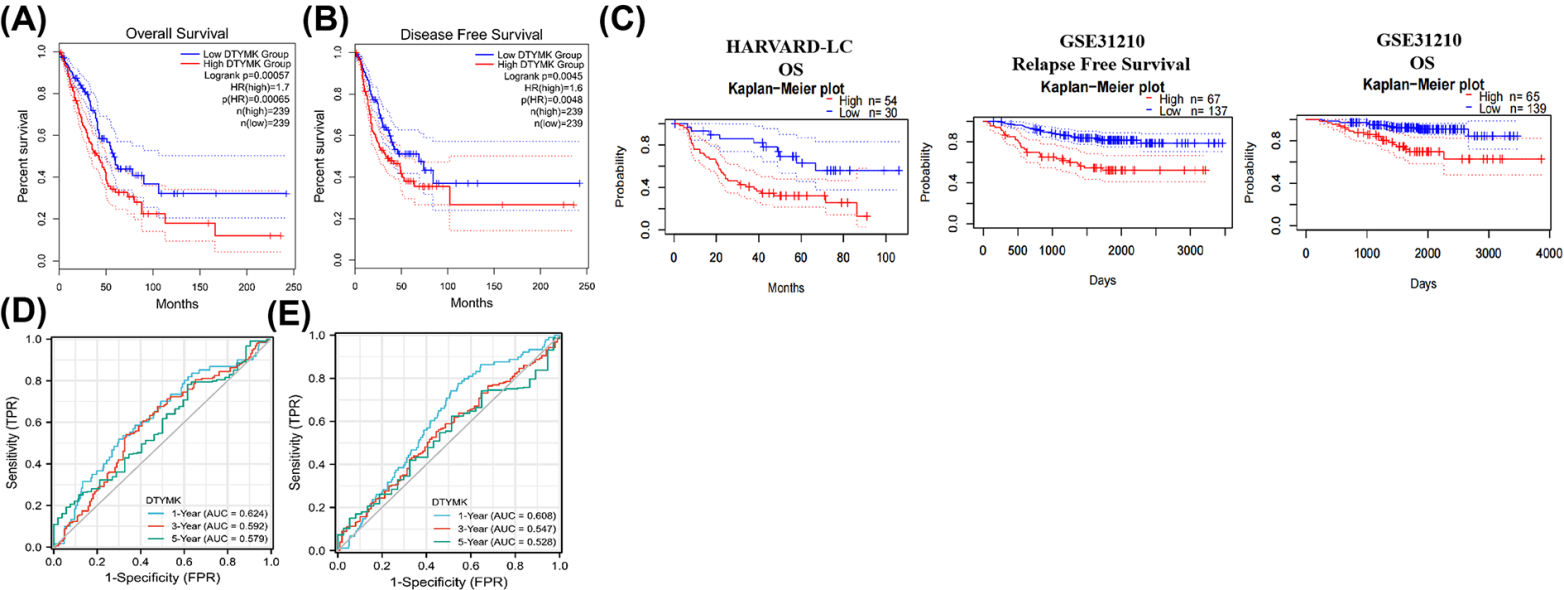
Association between DTYMK expression level and prognostic in LUAD (**A,B**) The survival curve indicated that LUAD patients with high expression level of DTYMK had shorter overall survival rate and disease-free survival rate, as revealed by TCGA database. (**C**) The survival curve indicated that LUAD patients with high expression level of DTYMK had shorter overall survival rate and disease-free survival rate, as revealed by GEO database (HARVARD-LC and GSE31210). (**D**) The time ROC analysis demonstrated the AUC for DTYMK in predicating risk score of OS in LUAD patients. (**E**) The time ROC analysis demonstrated the AUC for DTYMK in predicating risk score of DFS in LUAD patients.

With the use of data from the TCGA database, we examined the prognostic significance of DTYMK in LUAD patients. The median value of DTYMK was chosen as the cut-off point to classify LUSC patients into high- and low-expression groups. The findings illustrated that elevated DTYMK expression level was linked to a dismal OS rate and PFS rate (Figure 4A,B). In parallel, we further confirmed that the DTYMK expression level was negatively correlated with LUAD patients RFS and OS in the GEO database (HARVARD-LC and GSE31210), by utilizing the PrognoScan database (http://dna00.bio.kyutech.ac.jp/PrognoScan/index.html) (Figure 4C).

Subsequently, we conducted a time ROC analysis to compare the prediction performance and risk score of DTYMK for OS and PFS in LUAD. In the OS analysis, the DTYMK expression level could anticipate the LUAD patients’ prognoses over 1, 3, and 5 years and the area under the ROC curve (AUC) values were 0.624, 0.592, and 0.579, correspondingly (Figure 4D). In the PFS analysis, the AUC values were 0.608, 0.547, and 0.528, correspondingly (Figure 4E).

Finally, to investigate the potential relevance of DTYMK in LUAD from a prognostic standpoint, both univariate and multivariate Cox regression analyses were carried out. The findings from univariate analysis illustrated that T, N, M stage, pathologic stage, and DTYMK expression levels were considerably linked to OS rate in LUAD patients (Table 1). On the contrary, the multivariate analysis illustrated that only the T stage and N stage were substantially linked to OS rate in LUAD patients (Table 1).

**Table 1:** The univariate and multivariate Cox regression analysis of clinicopathological characters in LUAD

Characteristics	Total (*N*)	Univariate analysis		Multivariate analysis	
		Hazard ratio (95% CI)	*P* value	Hazard ratio (95% CI)	*P* value
T stage	523				
T1&T2	457				
T3&T4	66	2.317 (1.591–3.375)	**<0.001**	2.081 (1.373–3.152)	**<0.001**
N stage	510				
N0	343				
N1&N2&N3	167	2.601 (1.944–3.480)	**<0.001**	2.243 (1.587–3.172)	**<0.001**
M stage	377				
M0	352				
M1	25	2.136 (1.248–3.653)	**0.006**	1.456 (0.812–2.609)	0.207
DTYMK	526				
Low	262				
High	264	1.697 (1.263–2.279)	**<0.001**	1.331 (0.931–1.903)	0.117

### Expression profile of DTYMK in single cell analysis and its link to the functional state in LUAD

**Figure 5:**
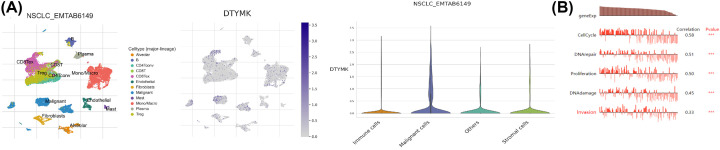
Expression profile of DTYMK in single-cell analysis and its link to the functional state in LUAD (**A**) GEO database EMTAB6149 demonstrated the DTYMK distribution in different cell types. (**B**) GSE69405 uncovered the association among DTYMK expression and cell cycle, DNA damage, DNA repair, invasion, and proliferation in LUAD.

Single-cell transcriptome sequencing is a crucial tool for assessing distinct cancer cells, stromal cells, endothelial cells, and immune cells due to the complexity of tumor cells. We used TISCH website (http://tisch.comp-genomics.org/home/) to analyze DTYMK expression distribution in tumor cells. The GEO database EMTAB6149 revealed that DTYMK expression was mainly distributed in malignant cells, when compared with its expression in immune cells, stromal cells and other cells (Figure 5A).

To validate the expression of DTYMK in single cells and its association with the functional state of tumors, the CANCER SEA website (http://biocc.hrbmu.edu.cn/CancerSEA/) was accessed. As revealed by data from GSE69405, the level of DTYMK expression was shown to have a substantial link to the cell cycle, DNA damage, DNA repair, invasion, and proliferation (Figure 5B).

### Analysis of enrichment pathways in LUAD together with the co-expression network of the DTYMK

**Figure 6:**
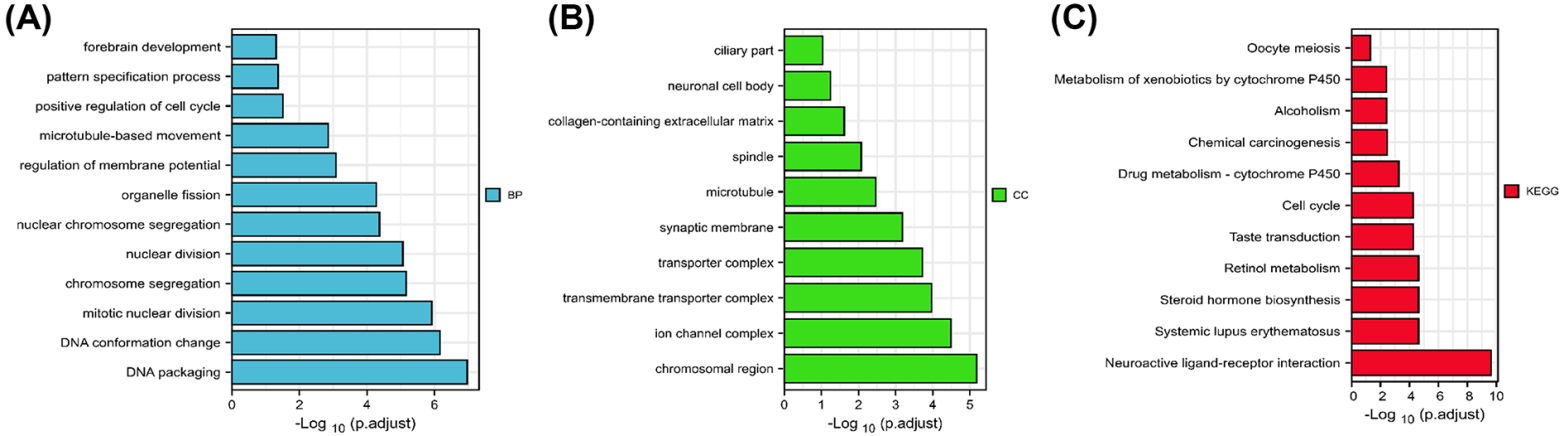
Co-expression network of DTYMK and enrichment pathway analysis in LUAD (**A**) GO_ BP of DTYMK correlated genes in LUAD. (**B**) GO_CC of DTYMK correlated genes in LUAD. (**C**) KEGG enrichment analysis of DTYMK correlated genes in LUAD.

Premised on the findings presented above, DTYMK may act as an oncogene in LUAD and may be a useful biomarker for determining the patients’ prognoses. We then performed a functional enrichment analysis of the DTYMK. Annotation using the biological process (BP) term demonstrated that genes that were co-expressed with DTYMK were mostly implicated in organelle fission, nuclear division, regulation of membrane potential, DNA conformation change, chromosome segregation, pattern specification process, mitotic nuclear division, positive regulation of cell cycle, and DNA packaging (Figure 6A). The cellular component (CC) analysis revealed that DTYMK were enriched dominantly in synaptic membrane, chromosomal region, neuronal cell body, microtubule, ciliary part, transmembrane, transporter complex, transporter complex, ion channel complex, collagen-containing extracellular matrix, and spindle (Figure 6B). The findings from the Kyoto Encyclopedia of Genes and Genomes (KEGG) pathway analysis highlighted a predominant enrichment of genes that were co-expressed in homologous recombination, cell cycle, DNA replication, and spliceosome, among other pathways (Figure 6C).

### Validation of DTYMK expression levels in LUAD tissues and cell lines

**Figure 7:**
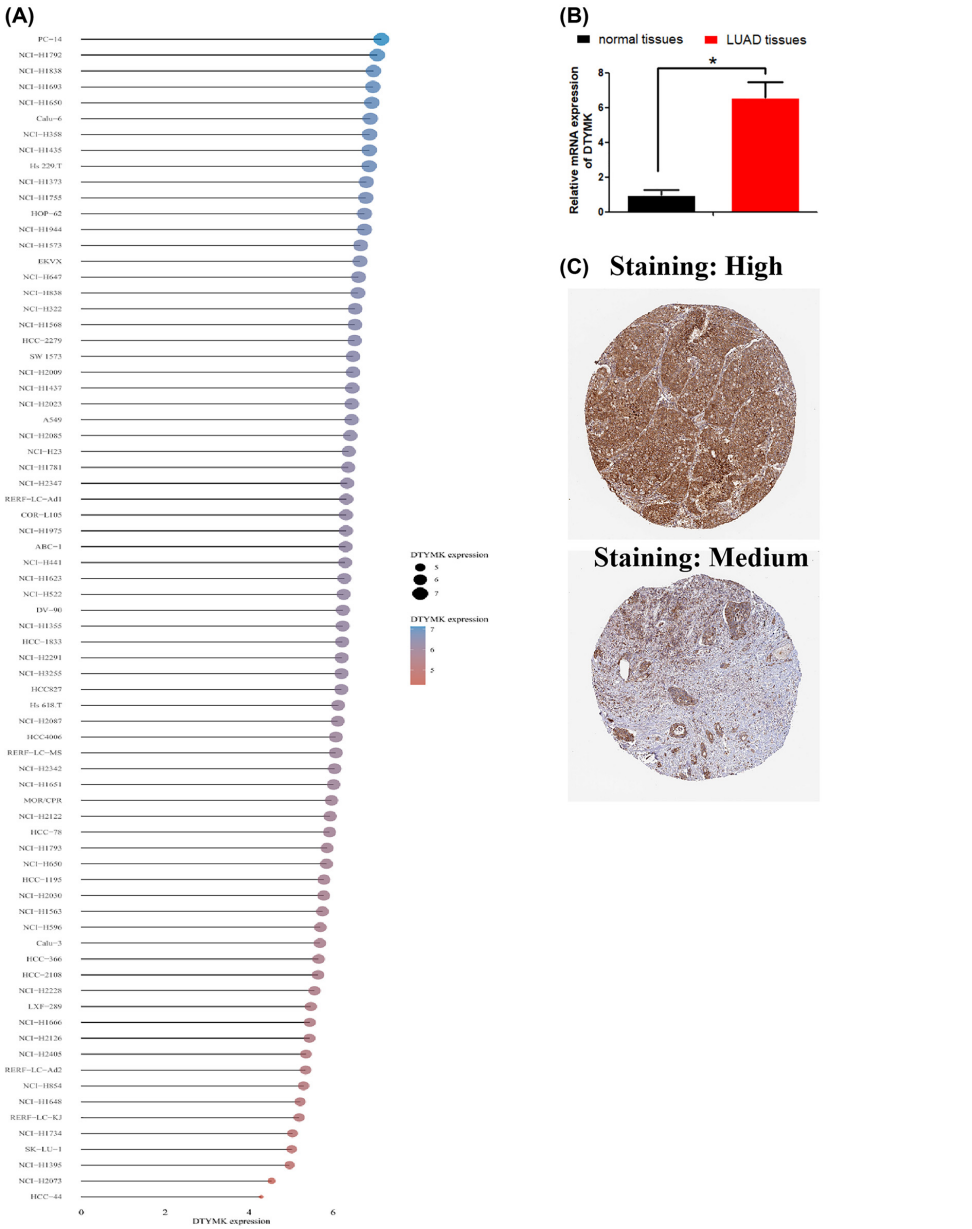
Validation of DTYMK expression levels in LUAD tissues and cell lines (**A**) The mRNA expression levels of DTYMK were demonstrated in LUAD cell lines by using of CCLE database. (**B**) RT-PCR assay was used to examine DTYMK mRNA expression levels in LUAD tissues and non-cancer tissues, respectively. (**C**) DTYMK protein expression levels were demonstrated in LUAD tissues by using The Human Protein Atlas database. *P<0.01, ***P<0.001.

We found by using CCLE database that the mRNA expression levels of DTYMK were substantially elevated in LUAD cell lines (Figure 7A). We confirmed that the DTYMK mRNA expression levels were elevated in LUAD in contrast with normal lung samples, as revealed by the RT-PCR assay (Figure 7B). With the use of The Human Protein Atlas database, we discovered that DTYMK protein expression levels were increased in LUAD samples. In addition, DTYMK was mainly distributed in cytoplasmic and membranous (Figure 7C).

### Inhibition of DTYMK decreased LUAD cell lines proliferation

The above findings suggested that DTYMK might be implicated in the proliferation and invasion of cells. We additionally performed a biological function assay to analyze DTYMK’s function in LUAD cell lines.

**Figure 8:**
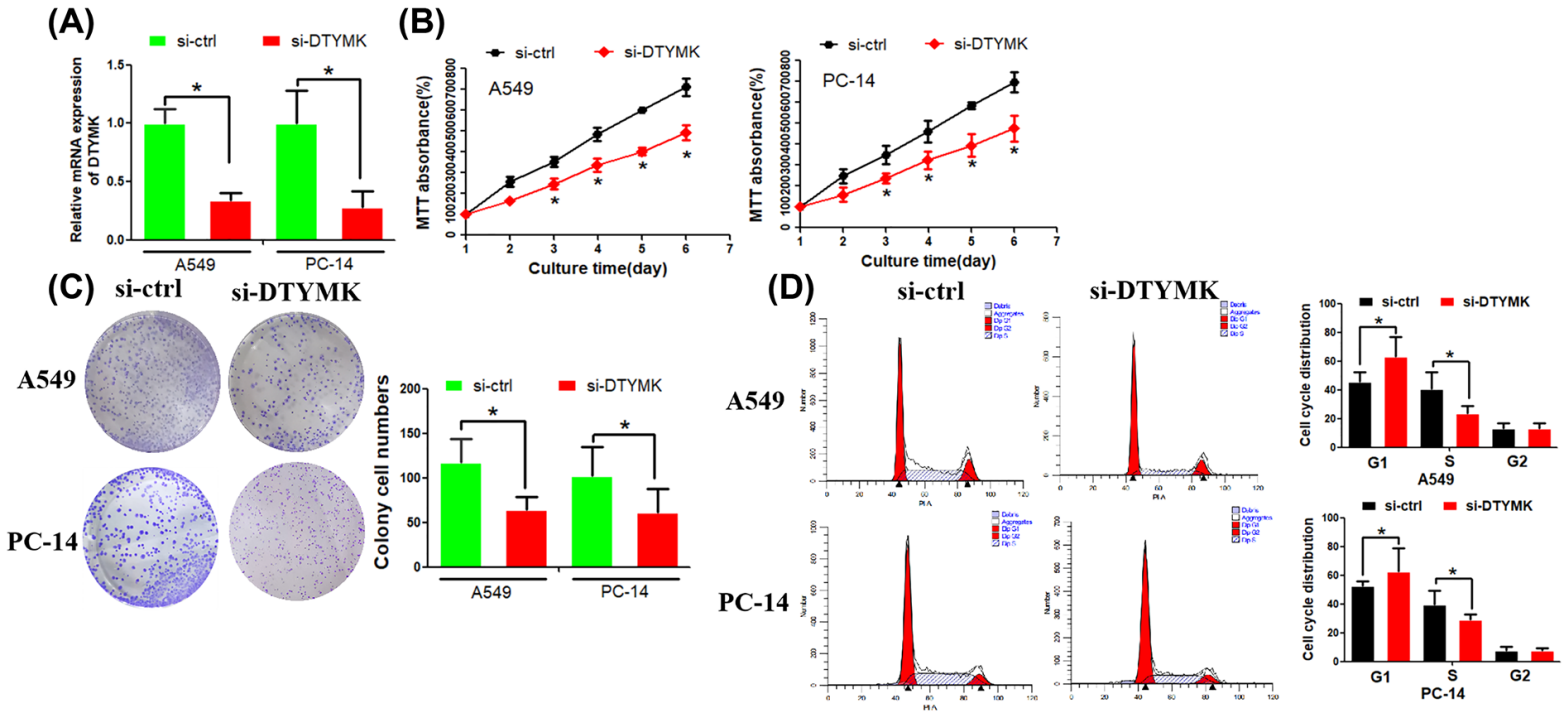
Inhibition of DTYMK decreased LUAD cell lines proliferation ability (**A**) RT-PCR assay was used to examine the efficiency of DTYMK down-regulation. (**B**) MTT assay was used to examine LUAD cell proliferation rate. (**C**) Colony formation assay revealed the DTYMK down-regulation’s effect on LUAD cells. (**D**) The flow cytometry assay was used to examine cell cycle distribution. *P<0.01.

DTYMK expression was suppressed in A549 and PC-14 cell lines by si-RNA that targeted the DTYMK sequence (Figure 8A). The proliferative capacity of A549 and PC-14 cells was remarkably suppressed when DTYMK was down-modulated, as illustrated by the MTT assay (Figure 8B). Colony formation assay revealed that A549 and PC-14 cells formed fewer and smaller colonies when DTYMK was inhibited (Figure 8C). These data suggested that DTYMK promoted LUAD cell proliferation. We then asked whether DTYMK affected cell cycle distribution. The flow cytometry analysis illustrated that the duration of the G0/G1 phase was prolonged, whereas the S-phase duration was shortened following DTYMK knockdown (Figure 8D).

### Inhibition of DTYMK increased CDDP sensitivity in LUAD cells

**Figure 9:**
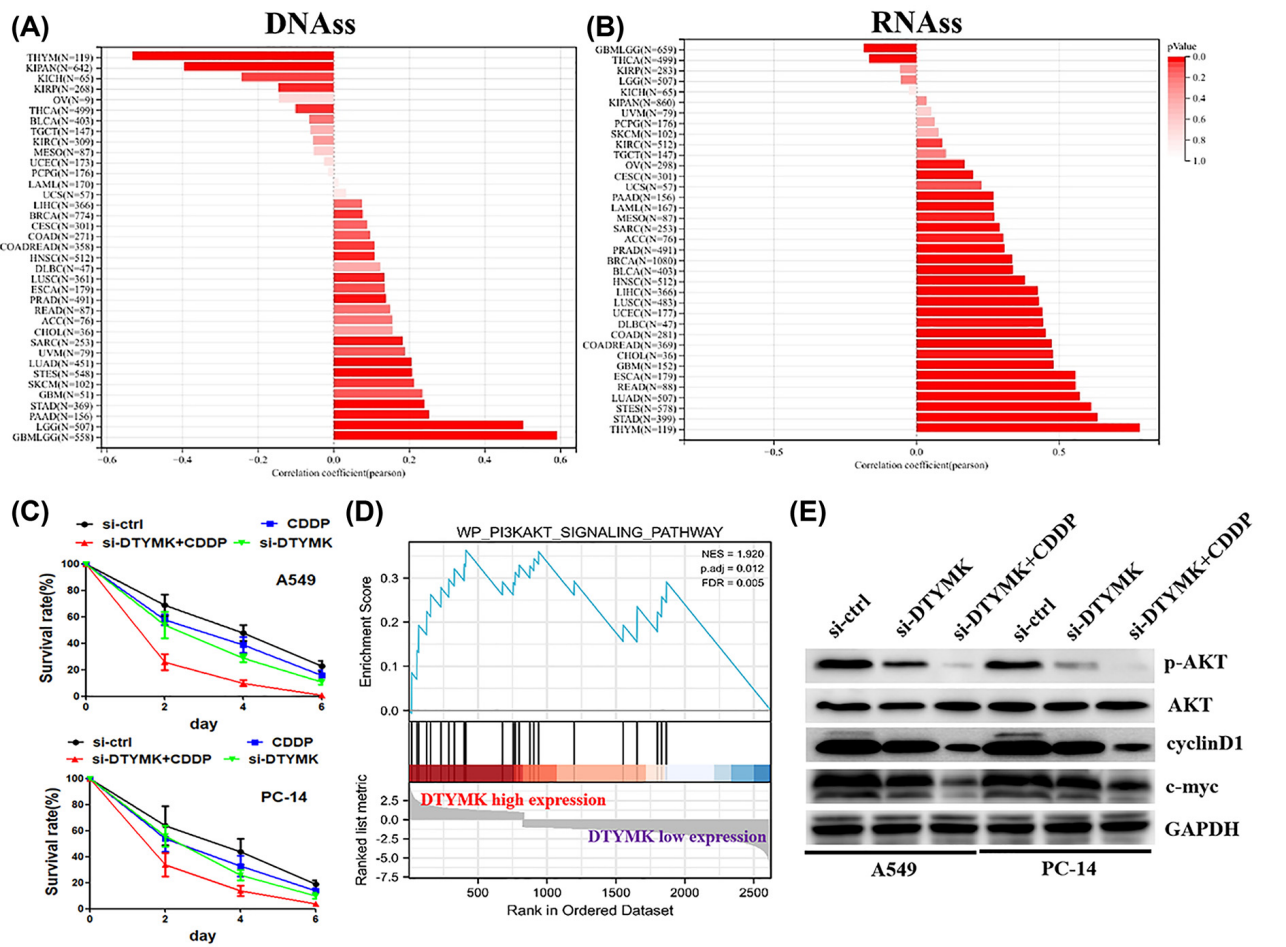
Inhibition of DTYMK increased CDDP sensitivity in LUAD cells (**A,B**) Correlation matrix between DTYMK geneexpression and cancer stemness scores DNAss and RNAss. (**C**) MTT assay was used to examine cell survival rate under the treatment of si-DTYMK, CDDP, or si-DTYMK+CDDP. (**D**) GSEA analysis revealed that PI3K/AKT signaling pathway was highly enriched in DTYMK-high samples. (**E**) Western blot assay was used to examine protein expression levels.

In the progression of cancer, tumor cells may eventually lose their differentiated phenotype and gain characteristics similar to those of progenitor and stem cells. RNA stemness score (RNAss), which is premised on mRNA expression, can be utilized to quantify the tumorstemness whereas DNA methylation pattern (DNAss) can be utilized to quantify DNA stemness [13]. We investigated whether DTYMK genes had an association with the stemness of tumors, by using DNAss and RNAss analysis. The DTYMK expression was shown to have varying degrees of links to DNAss and RNAss among diverse kinds of cancer (Figure 9A,B). Because the above findings revealed that DTYMK genes were commonly linked to stem cell-like characteristics, we evaluated whether inhibiting DTYMK influenced the sensitivity of LUAD cell lines to CDDP. As expected, down-regulation of DTYMK increased CDDP sensitivity in LUAD cells (Figure 9C).

### Inhibition of DTYMK decreased PI3K/AKT signaling pathway in LUAD cell lines

With the use of GSEA analysis, we found that PI3K/AKT signaling pathway was highly enriched in DTYMK-high samples compared with DTYMK-low samples (Figure 9D). We then further analyzed whether DTYMK indeed affected PI3K/AKT signaling pathway by performing biological analysis. Western blotting illustrated that down-modulation of DTYMK decreased the expression levels of p-AKT, instead of total AKT. In addition, down-regulation of DTYMK also decreased the expression levels of PI3K/AKT signaling pathway downstream targets (e.g. cyclinD1 and c-myc) (Figure 9E). Taken together, our data revealed that inhibition of DTYMK impaired PI3K/AKT signaling pathway activity.

## Discussion

Because of rapid advances in medical technology and the persistent research efforts of oncologists, the diagnosis, monitoring, and treatment of oncological illnesses are presently becoming more scientific, universal, and individualized. DTYMK is a nuclear deoxythymidylate kinase that is responsible for catalyzing the deoxy-TMP phosphorylation process. It is the enzyme that catalyzes the final process in the pathways that produce deoxyribonucleoside triphosphate (dTTP) and is extensively expressed in all tissues. Studies reported in previous decades have illustrated that DTYMK is likely to have a function in determining a grim prognosis in patients with non-small cell lung cancer (NSCLC), and it might be a viable treatment target for these patients. However, neither the biological function that DTYMK plays nor the mechanisms behind it is fully known at this time.

This is the first study of its kind to comprehensively examine DTYMK in pan-cancer in such depth. NSCLC is one of the most prevalent malignancies across the globe. LUAD represents 40% of all cases of NSCLC. Our analysis demonstrated that DTYMK expression level was significantly associated with LUAD patients’ DFS and OS. We thus further study DTYMK biological function in LUAD in detail.

Our investigation centered on the significant role that the DTYMK gene plays in the progression of a variety of malignancies. To do this, we meticulously searched through the data on a wide range of cancer types and enrolled a large sample size. Initially, we found the aberrant expression of DTYMK in a variety of malignancies. Through the use of the Cox regression model, we identified distinct expressions of DTYMK in a variety of survival markers; these findings revealed DTYMK as a potentially useful prognostic component of specific cancers.

In the field of oncology, immunotherapy is now a very popular subject of discussion. Tumor-infiltrating lymphocytes, TMB, and MSI status perform fundamental functions in eliciting the responsiveness to immunotherapy and determining the clinical outcomes in cancers. In our research, we discovered that DTYMK was able to reliably activate all six different kinds of immune cells (neutrophils, CD8+ T cells, macrophages, dendritic cells, CD4+ T cells, and B cells) in most kinds of cancer, except in BLCA and CHOL. The key immune checkpoints (CD274, CTLA-4, HAVCR2, LAG3, PDCD1, PDCD1LG2, SIGLEC15, and TIGIT) also had a significant relationship with DTYMK expression in approximately 20 kinds of cancers, except in MESO, PCPG, and UCS. Certain gene mutations may be used to anticipate patients’ prognoses and the way they will respond to therapy. Higher somatic TMB and MSI were shown to be linked to enhanced efficacy with immunotherapy as well as favorable OS survival for the majority of cancer histology. The upstream causes of MSI were identified as mutations in mismatch repair genes as well as an impaired function in those genes. In this work, DTYMK expression level was positively linked to TMB in approximately 20 kinds of cancers but negatively associated with TMB in THYM. Additionally, we discovered a positive link between DTYMK expression level and MSI status in UCEC, STAD, PRAD, LIHC, and KIRC. All of these findings indicated the possibility that DTYMK may promote tumor growth by increasing MSI and TMB via modulating genes implicated in mismatch repair. High TMB and MSI-H status may be heavily infiltrated by immune cells making them amenable to respond to immune checkpoint inhibitors. Given this conception, the cancer types with high DTYMK expression level may benefit from treatment of immune checkpoint inhibitors.

Since single-cell transcriptome sequencing and KEGG pathway analysis illustrated that DTYMK expression was substantially linked to cell cycle and proliferation in NSCLC. The functional assays confirmed that inhibition of DTYMK decreased LUAD cell line growth and affected cell cycle distribution.

Cancer stem-like cells (CSCs) could originate from multiple sources, such as progenitor cells or long-lived stem cells, or through the process of dedifferentiation in non-stem cancer cells, which facilitates these cells to transform into CSCs by dysregulating relevant signaling pathways. CSCs are the main contributor to treatment-induced medication resistance, and they are responsible for the advancement of cancer owing to their ability to both self-renew and invade surrounding tissues. In the current analysis, we examined the expression of DTYMK with characteristics similar to those of stem cells characteristics using RNAss and DNAss as our measuring tools. The expression level of DTYMK was shown to have a positive correlation with DNAss and DNAss in almost all forms of cancers, including LUAD. Importantly, we revealed that down-regulation of DTYMK increased CDDP sensitivity in LUAD cells. According to these findings, DTYMK could be linked to enhanced cancer cells’ resistance to chemotherapeutic treatments.

To explore the mechanism of how DTYMK regulated LUAD cell proliferation and invasion, we performed a GSEA analysis. It was found that PI3K/AKT signaling pathway was highly enriched in DTYMK-high samples. Further investigation confirmed that down-regulation of DTYMK indeed inhibited PI3K/AKT signaling pathway activity.

In summary, our research was the first to conduct a complete analysis of DTYMK in pan-cancer. We discovered that the expression level of DTYMK was elevated in a variety of malignancies and was correlated adversely with OS. According to the results of our validation experiment, the expression level of DTYMK was considerably elevated in LUAD samples as opposed to the matching non-tumor samples. This finding implies that DTYMK might be used as a biological marker of LUAD. DTYMK might perform an integral function in LUAD advancement by engaging in cell cycle progression-induced proliferation of LUAD cells.

## Supplementary Material

Supplementary Figure S1 and Tables S1-S4

## Data Availability

The data used to support the findings of the present study are available from the corresponding author upon request.

## Abbreviations

CCLE: Cancer Cell Line Encyclopedia
CSC: cancer stem-like cell
dTMP: deoxythymidine-5′-monophosphate
DTYMK: deoxythymidylate kinase
LUAD: lung adenocarcinoma
MSI: microsatellite instability
NSCLC: non-small cell lung carcinoma
TCGA: The Cancer Genome Atlas
TMB: tumor mutation burden
