# Supplementary figures and images for: Comprehensive analysis of DTYMK in pan-cancer and verification in lung adenocarcinoma
Source: Biosci Rep. 2022 Oct 18;42(10):BSR20221170. doi: 10.1042/BSR20221170 (PMC9583767; doi:10.1042/BSR20221170)

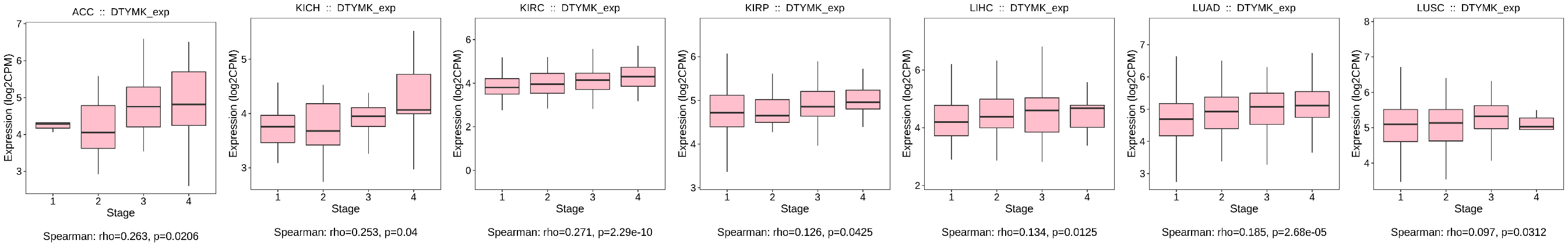

Supplement: Supplementary Figure S1 and Tables S1-S4 [file BSR-2022-1170_supp.zip › Supplementary figure 1.tif]
